# Challenging the common practice of intravenous fluid administration: Viewpoint on the need for an enteral fluid therapy trial in critically ill patients

**DOI:** 10.1007/s00508-024-02335-3

**Published:** 2024-03-08

**Authors:** Nikolaus Keil, Vincent Rathkolb, Maximilian Waller, Simon Krenn, Georg Hinterholzer, Wilfred Druml, Michael Hiesmayr, Sabine Schmaldienst, Manfred Hecking

**Affiliations:** 1 First Medical Department, Klinik Favoriten, Vienna, Austria; 2 AIT Austrian Institute of Technology GmbH, Center for Health & Bioresources, Medical Signal Analysis, Vienna, Austria; 3 Department of Medicine III, Division of Nephrology & Dialysis, Medical University of Vienna, Vienna, Austria; 4 Department of Anesthesiology, Intensive Care Medicine and Pain Medicine, Division of Cardiac Thoracic Vascular Anesthesia and Intensive Care Medicine, Medical University of Vienna, Vienna, Austria

## Viewpoint

Intravenous fluid therapy is frequently and ubiquitously used across clinical disciplines, and it is considered indispensable for treating various serious diseases [1]. Although this practice is currently widely used, the choice and amount of fluid, the timing, and the method of application have long been controversial [2]. Most volume substitution agents in current use were approved at a time when only minimal, if any, safety data requirements were necessary to gain approval of therapeutics. Ringer’s lactate, normal saline and human albumin required, at most, evidence that they did not cause acute toxicity or hemolysis [3]. To date, there are no prospective intervention studies comparing different volumes for initial resuscitation in patients with septic shock. This was shown in the 2021 Surviving Sepsis Campaign [4] that recommends treating patients with sepsis and septic shock with intravenous administration of 30 ml/kg body weight of crystalloid fluid within the first 3 h. This only weak recommendation with low quality evidence is based solely on observational data and a single retrospective analysis of patients admitted to the emergency department [5]. In contrast to the lack of evidence on the correct amount of fluids for volume substitution, the evidence regarding fluid overload is abundant. The SOAP study found that a positive fluid balance in patients with sepsis was the second strongest prognostic factor for mortality, after old age [6]. Other studies have demonstrated that restrictive intravenous fluid regimens, compared to a liberal approach, resulted in less acute kidney injuries in septic shock [7], better pulmonary outcomes in acute respiratory distress syndrome [8] and fewer postoperative complications after visceral surgery [9]. In addition, early administration of a bolus of intravenous fluid was shown to significantly increase mortality in critically ill children, compared to treatment without a bolus, in resource-limited settings [10]. Extensive fluid therapy can lead to hospital-acquired, generalized, interstitial edema in critically ill patients [11] and an increase in diffusion distance and tissue pressure, which results in poor tissue perfusion. These pathophysiological conditions are unlikely to be unique to patients with severe diseases. The infusion of balanced salt solutions in healthy volunteers with a normal capillary leak index led to interstitial fluid accumulation, including a reduction in intracellular volume [12]. Furthermore,, the infusion of normal saline resulted in reduced renal blood flow and cortical tissue perfusion in healthy individuals [13]. These results suggest that even in people with healthy renal and cardiac functions, intravenous fluid intake, rather than the disease alone, may be a major cause of adverse fluid retention. In contrast to earlier findings, two recent large randomized trials, the CLOVERS trial [14] and the CLASSIC trial [15], investigated the effect of liberal versus restrictive fluid management in patients with septic shock. Neither trial demonstrated a significant difference in mortality rates between the two groups. This discrepancy in clinical findings could be attributable to a paradigm shift over the last decades from extensive to more restrictive fluid management in critically ill patients. For instance, a 2013 databank analysis showed an average fluid administration of 4.4 l to 23,513 patients with septic shock in the first 24 h [16]. In contrast, the CLASSIC trial administered 1.7–3.8 l to 1554 patients over a median period of 5 days [15] and 1563 patients in the 2023 CLOVERS trial received between 1.2 and 3.4 l of intravenous fluids in the initial 24 h [14]. The shift towards more restrictive fluid management as a standard of care in intensive care units (ICU), and the resulting smaller differences in treatment approaches across these studies, might contribute to the lack of observable clinical benefits in the context of increasingly restrictive fluid management strategies [17].

Another disorder associated with the liberal use of intravenous fluid administration is ICU-acquired hypernatremia. This common electrolyte disturbance in the ICU often results from excessive intravenous administration of sodium-rich fluids or the loss of free water and was shown to be an independent risk factor for mortality [18, 19]. This is particularly relevant in patients with compromised renal function or altered mental states, such as those sedated or intubated [18]. A more physiological approach would be to administer fluids enterally. In the gut, the absorption of glucose, electrolytes, and water is autoregulated by homeostatic mechanisms, mainly in the small intestine [20]. These mechanisms include the glucose-sodium symporter, the Na^+^/H^+^ antiporter, and epithelial Na^+^ channels. Water absorption occurs both paracellularly and transcellularly, and it is coupled to the transport of water-soluble substances [20]. There are currently only few clinical trials investigating enteral fluid replacement, but available data are positive: a 2018 meta-analysis including 4 randomized controlled trials (RCT) with 538 patients found oral hydration as effective as intravenous hydration in preventing contrast-induced nephropathy [21]. A 2015 randomized comparative trial showed no difference in preventing disease-specific outcomes and mortality in 49 patients with acute pancreatitis who underwent nasojejunal or intravenous fluid resuscitation [22]. A secondary analysis of a 2020 multicenter RCT showed no inferiority of oral versus intravenous fluid therapy in 505 children who required nasal high-flow therapy for bronchiolitis. [23]. A 2004 meta-analysis of 16 RCTs found equal efficacy of oral fluid administration compared with intravenous fluid therapy in 1545 children with gastroenteritis, with a significant reduction in length of hospital stay and fewer serious adverse events in the oral group [24].

However, there are currently no prospective RCTs comparing enteral and intravenous fluid administration in patients requiring intensive care.

## Trial proposal

**Fig. 1:**
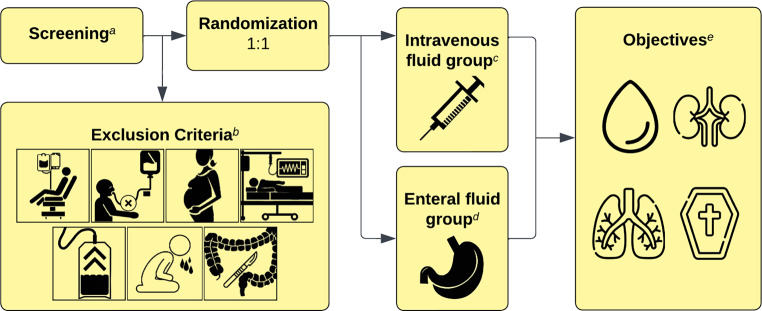
Study design. ^a^ Eligibility criteria for screening: intubation within 72 h and age over 18 years old. ^b^ Exclusion criteria: kidney replacement therapy before intubation, inability to receive enteral nutrition, pregnancy, postoperative patients with consecutive intensive care admission, expected required fluid > 4 ml/kg/h for at least 24 h, evidence of gastrointestinal disease, abdominal surgery in the last 3 months. ^c^ Anticipated volume of intravenously applied fluids in the intravenous group: 2211 mL/24 h. ^d^ Anticipated volume of intravenously applied fluids in the enteral group: 1613 mL/24 h. Anticipated volume of intravenously applied fluid in the enteral group: 1376 mL/24 h. ^e^ Primary objectives: incidence and extent of regurgitation. Secondary objectives: serum sodium levels, serum osmolality, renal function parameters, days on ventilation, and mortality

In the absence of prospective data, we find it is high time to evaluate the safety and feasibility of enteral fluid administration compared to intravenous fluid administration in critically ill patients. A prospective, randomized, parallel group, open-label, exploratory study would test the hypothesis that enteral fluid therapy via a nasogastric tube, is as safe and feasible as intravenous fluid therapy, for patients that require intensive care. Inclusion criteria would be intubation within 72 h and age over 18 years. Exclusion criteria would be kidney replacement therapy before intubation, inability to receive enteral nutrition, pregnancy, postoperative patients with consecutive intensive care admission, expected required fluid > 4 ml/kg/h for at least 24 h, evidence of gastrointestinal disease and abdominal surgery in the last 3 months. Eligible patients from the ICU would need to be randomized to either standard practice (intravenous fluid administration with no enteral fluid administration other than enteral nutrition) or the test practice (enteral fluid administration). In the enteral fluid group, enteral fluid administration would need to be the primary mode of administration. If needed, this group could also receive intravenous fluids at the discretion of the physician but this would remain the secondary administration route. The primary enteral fluid would be tap water and the intravenous fluid of choice would be balanced multielectrolyte solutions. The volume of fluid administered in each treatment group would be determined by the treating physician based on clinical decision-making, without prespecification in the protocol. The primary objective would be to evaluate safety and feasibility by comparing the incidence of regurgitation (defined as > 500 mL in 24 h) and the extent of daily regurgitation. Secondary endpoints would include clinical and laboratory parameters, such as fluid status, serum sodium levels, serum osmolality, renal function parameters, days on ventilation, and mortality that should be collected in an exploratory fashion (Fig. 1). Fluid status would be evaluated by the supervising physician during daily rounds with a standardized protocol using clinical and sonographic findings. Furthermore, trained study personnel would perform bioimpedance spectroscopy measurements every 48 h. The intervention period would be from the time of inclusion until the time of extubation or death. The assessment of the intervention’s tolerability and safety would involve monitoring gastrointestinal symptoms including absent bowel sounds, regurgitation > 500 mL, gastrointestinal bleeding, diarrhea (liquid stools > 3 times/day) and bowel distension assessed by ultrasound. Patients exhibiting more than three of these symptoms within 24 h would be excluded for safety considerations. All adverse events, such as ventilator-associated pneumonia or aspiration would be documented and reported by the study personnel.

## Conclusion

As intravenous fluid administration is common in hospitals, especially in intensive care units, where independent drinking is not usually possible, adverse effects such as hypernatremia are frequent, particularly in the context of renal injury [18]. Other risks include prolonged ICU stay, worsening respiratory function and salt retention [7, 8, 18]. In healthy individuals, fluid intake usually occurs through hypotonic oral fluids, making intravenous fluid replacement with isotonic or partially hypertonic fluids nonphysiological. A more physiological approach to therapy, in our opinion, is worth pursuing. The presented outline of an exploratory study evaluating feasibility might be used for follow-up studies to calculate an appropriate sample size and to estimate an effect size of treatment differences between enteral and intravenous fluid administration. Study staff, familiar with both enteral and intravenous fluid administration procedures and physicians may still opt for intravenous fluid administration in the enteral study group, if necessary, which would ensure safety in both study groups. This study would offer potential benefits to participants due to enhanced fluid therapy monitoring compared to standard ICU practices. Such a study’s strength lies in its novelty, being the first to assess the feasibility of enteral fluid administration in critically ill patients. Overall, this research could offer new insights into fluid administration practices in critical care settings.
